# Mitochondrial PGAM5−Drp1 signaling regulates the metabolic reprogramming of macrophages and regulates the induction of inflammatory responses

**DOI:** 10.3389/fimmu.2023.1243548

**Published:** 2023-09-12

**Authors:** Bo-Ram Bang, Haruka Miki, Young Jun Kang

**Affiliations:** ^1^ Department of Immunology and Microbial Science, The Scripps Research Institute, La Jolla, CA, United States; ^2^ Division of Immune Regulation, La Jolla Institute for Immunology, La Jolla, CA, United States; ^3^ Department of Rheumatology, Institute of Medicine, University of Tsukuba, Tsukuba, Japan; ^4^ Molecular Medicine Research Institute, Sunnyvale, CA, United States

**Keywords:** innate immunity, macrophages, inflammatory response, mitochondria, metabolism, signaling/signaling pathways

## Abstract

Macrophages play a critical role in the regulation of inflammation and tissue homeostasis. In addition to their vital functions for cell survival and physiology, mitochondria play a crucial role in innate immunity as a platform for the induction of inflammatory responses by regulating cell signaling and dynamics. Dynamin-related protein 1 (Drp1) plays a role in the induction of inflammatory responses and the subsequent development of various diseases. PGAM5 (phosphoglycerate mutase member 5) is a mitochondrial outer membrane phosphatase that dephosphorylates its substrate, Drp1. Previous studies showed that PGAM5 regulates the phosphorylation of Drp1 for the activation of NKT cells and T cells. However, it is not clear how PGAM5 regulates Drp1 activity for the induction of inflammation in macrophages. Here, we demonstrate that PGAM5 activity regulates the dephosphorylation of Drp1 in macrophages, leading to the induction of proinflammatory responses in macrophages. In TLR signaling, PGAM5 regulates the expression and production of inflammatory cytokines by regulating the activation of downstream signaling pathways, including the NF-κB and MAPK pathways. Upon LPS stimulation, PGAM5 interacts with Drp1 to form a complex, leading to the production of mtROS. Furthermore, PGAM5-Drp1 signaling promotes the polarization of macrophages toward a proinflammatory phenotype. Our study further demonstrates that PGAM5-Drp1 signaling promotes metabolic reprogramming by upregulating glycolysis and mitochondrial metabolism in macrophages. Altogether, PGAM5 signaling is a linker between alterations in Drp1-mediated mitochondrial dynamics and inflammatory responses in macrophages and may be a target for the treatment of inflammatory diseases.

## Introduction

Macrophages play a critical role in inflammation, metabolism, wound repair, host defense against microbial infection, and tumor surveillance. Emerging evidence suggests that cell metabolism plays a role in determining the phenotypes of macrophages. M1 macrophages that express proinflammatory cytokines and inducible nitric oxide synthase (NOS2) are largely dependent on glycolysis for ATP generation, partly due to the disrupted TCA cycle and fatty acid synthesis (1–3). In contrast, M2 macrophages require oxidative phosphorylation and fatty acid oxidation for anti-inflammation gene expression (4–6).

Mitochondria are a major source of energy for physiological processes and incredibly dynamic organelles (7). Additionally, they are also involved in the regulation of programmed cell death, such as apoptosis and necroptosis. Recently, emerging evidence suggests a role for mitochondria as a platform for innate immune signaling pathways (8). Proinflammatory macrophage responses are initiated by the recognition of pathogen-associated molecular patterns (PAMPs), leading to dynamic changes in macrophage metabolism, and the activation signals that regulate the electron transport chain and the TCA cycle to influence mitochondrial metabolism.

Mitochondria morphology changes dynamically by coordinated fission and fusion cycles, which are crucial for many cellular processes including immune responses, cell cycle, cell death, and the maintenance of mitochondrial quality (9). Mitochondrial dynamics are controlled by several dynamin-related GTPases, which is essential for the fission and fusion of mitochondria (10). Accumulating data suggest that dynamin-related protein 1 (Drp1),one of the dynamin-related GTPases, plays a role in disease development (11–13), and Drp1 regulates the mitochondrial fission and subsequent inflammatory responses of macrophages (14, 15). Furthermore, PKCδ- or Stat2-dependent activation of Drp1 regulates the induction of proinflammatory responses in macrophages (16, 17), suggesting that Drp1 plays a crucial role in connecting alterations in mitochondrial dynamics and innate immunity.

PGAM5 (phosphoglycerate mutase 5) is a mitochondrial outer membrane phosphatase that dephosphorylates its substrate, Drp1 (10, 18). PGAM5 regulates the phosphorylation status of Drp1 for the regulation of NKT cell-mediated immune responses in liver inflammation and anti-tumor immunity (19), while PGAM5 functions as a phosphohistidine phosphatase for TCR-stimulated Ca^2+^ influx in T cells (20). However, it is not clear whether PGAM5 in macrophages regulates the pro-inflammatory responses and how PGAM5 contributes to the metabolic reprogramming and phenotype determination of macrophages.

In this study, we address these questions by providing compelling evidence that TLR-dependent PGAM5 activity regulates Drp1 phosphorylation in macrophages, leading to the induction of proinflammatory responses in macrophages. Our study further demonstrates that PGAM5-Drp1 signaling promotes metabolic reprogramming by regulating glycolysis and mitochondrial metabolism in macrophages.

## Materials and methods

### Mice

C57Bl/6 background WT mice were obtained from Jackson Laboratory, and *Ripk3* ^–/–^ mice were kindly obtained from Dr. Dixit (Genentech, Inc.). Sex- and age-matched (8 – 12 weeks of age) mice were used. Animal protocols were approved by the Institutional Animal Care and Use Committee.

### Endotoxin-induced septic shock in mice

C57Bl/6 mice (male, 8 – 10 weeks) were i.p. injected with 10 or 20 mg/kg Mdivi-1 or diluted DMSO in PBS for 2 h, followed by i.p. injection of 15 mg/kg LPS. Serum samples were collected at 1 or 2 hours after LPS injection, and the survival of mice was monitored.

### Reagents

LPS from *Escherichia coli* O111:B4 was obtained from List Biolabs and Sigma. Mdivi-1 was obtained from Enzo Life Sciences. Actinomycin D was purchased from Calbiochem. Phospho-ASK1 was purchased from Santa Cruz Biotechnology. Anti-GAPDH antibody was obtained from Chemicon. IκBα, phospho-p38α, phospho-JNK, phospho-ERK, Drp1, and phospho-Drp1 (Ser 637) antibodies were obtained from Cell Signaling Technology.

Control, MyD88 inhibitory peptide, and TRIF inhibitory peptide were purchased from IMGENEX. The amino acid sequences are; control peptide, RQIKIWFQNRRMKWKK; MyD88 inhibitor peptide, RQIKIWFQNRRMKWKKRDVLPGT; TRIF inhibitor peptide, RQIKIWFQNRRMKWKKFCEEFQVPGRGELH.

### Cell culture

HEK 293T cells and bone-marrow-derived macrophages (BMDM) were cultured in Dulbecco’s modified Eagle’s medium (DMEM) supplemented with 10% fetal bovine serum and antibiotics. Peritoneal macrophages were obtained from the peritoneal cavities of mice 3 days after intraperitoneal injection of 4% thioglycollate. BM cells obtained from the femurs of mice were cultured in M-CSF (10 ng/ml). Immortalized macrophages (iBMDMs) were generated by infecting BMDMs with retroviruses encoding v-*raf* and v-*myc* as previously reported (21).

### Flow cytometry

To assess the polarization of macrophages, LPS-treated macrophages were incubated for 24 hours. Cells were incubated with anti-mouse CD16/CD32 Ab, followed by cell surface staining with CD11b-FITC (clone M1/70, Thermo Fisher), F4/80-PerCPCy5.5 (clone BM8, Thermo Fisher), and CD11c-AF700 (clone N418, Thermo Fisher). After fixation and permeabilization, intracellular NOS2 was stained with NOS2-PE eF610 (clone CXNFT, Thermo Fisher).

To test the levels of mtROS, macrophages were incubated with MitoSOX (5 μM) and LPS for 12 hours and washed. Data from FACSCalibur (BD Biosciences) were analyzed with FlowJo software.

### Short-hairpin RNA plasmids and lentiviruses

Preparation of plasmids for shRNA targeting PGAM5 or Drp1 and recombinant lentivirus packaging were conducted as previously reported (19).

### Dual luciferase reporter assay

Control iBMDMs were transfected with NF-AT-, AP-1-, C/EBP-, or CREB-driven firefly luciferase plasmids, and pTK-RL *Renilla* plasmid by using Lipofectamine 3000 transfection reagent following the manufacturer’s protocol (Thermo Fisher). After 48 hours, cells were treated with LPS for 24 hours to measure the luciferase activity using the Dual-Luciferase Reporter Assay System (Promega). Activity of the *Renilla* luciferase was measured as the internal control. The fold induction of luciferase activity was calculated to the untreated cells.

### RNA-sequencing analysis

Total RNA was isolated using RNeasy kit (Qiagen) following the manufacture’s instruction. The purity and concentration of RNA samples were measured using Nanodrop Spectrophotomemter. Preparation of cDNA library and transcriptome sequencing was conducted by Novogene Co., LTD (Davis, CA). Differential gene expression was analyzed by iDEP (integrated Differential Expression and Pathway analysis) (22). Genes with |log2(FoldChange)| > 1 and adjusted p-value < 0.05 were considered as “differentially expressed”. RNA-Seq data have been deposited at GEO and are openly available in NCBI GEO at https://www.ncbi.nlm.nih.gov/geo, reference number GSE235306.

### Quantitative RT-PCR

Using total RNAs from macrophages, cDNA was prepared and quantitative PCR (qPCR) analysis was performed using PowerUp SYBR Green Master Mix (Thermo Fisher). Gene expression level was calculated by normalizing to *Gapdh* mRNA level. The sequences of primer sets are shown in **Table 1**.

**Table 1:** Primer sequences for quantitative PCR.

Gene	Forward (5’→ 3’)	Reverse (5’→ 3’)
*Il6*	GAGGATACCACTCCCAACAGACC	AAGTGCATCATCGTTGTTCATACA
*Il23*	GGTGGCTCAGGGAAATGT	GACAGAGCAGGCAGGTACAG
*Tnf*	ATGAGCACAGAAAGCATGA	AGTAGACAGAAGAGCGTGGT
*Il1b*	TGGACCTTCCAGGATGAGGACA	GTTCATCTCGGAGCCTGTAGTG
*Nos2*	CCCTTCCGAAGTTTCTGGCAGCA	GGCTGTCAGAGAGCCTCGTGGCTTT
*Gapdh*	TCAAGAAGGTGGTGAAGCAG	TCGCTGTTGAAGTCAGAGGA

### Measurement of mRNA stability

To analyze *Tnf* or *Il6* mRNA stability, macrophages were stimulated with LPS, followed by actinomycin D (ActD, 10 µg/ml) addition to block transcription. Total RNAs were extracted from cells for qRT-PCR. The mRNA level of cells without ActD was set as 100%. The percentage of mRNA abundance in ActD-treated cells at different times was calculated.

### Immunoprecipitation and immunoblotting

Macrophages were washed and incubated at 4°C for 30 min in lysis buffer containing protease inhibitor cocktail (Roche), 1 mM DTT, and 1 mM PMSF. After 30 min at 4°C, the cell suspensions were centrifuged at 12,000 rpm for 10 min at 4°C. Protein concentrations were determined by the BCA method (Thermo Fisher). Cell lysates were incubated with isotype (goat Ig) or anti-PGAM5 Abs, and further incubated with Protein G-agarose. Immunoprecipitates were washed with lysis buffer and subjected to SDS-PAGE.

Whole cell lysates were separated with SDS-PAGE, and proteins were transferred to membranes. Immunoblotting was performed with the appropriate primary and HRP-conjugated secondary antibodies and visualized by chemiluminescence.

### Measurement of cytokine concentrations

TNF and IL-6 levels in sera or culture supernatants were measured by ELISA according to the manufacturer’s protocol (Thermo Fisher).

### Intracellular succinate or triglyceride quantification

Succinate or triglyceride levels in macrophages were determined using Succinate Assay Kit or Triglyceride Assay Kit (Abcam).

### Extracellular flux analysis

Metabolic changes were monitored by an extracellular flux analyzer XFe96 (Seahorse Bioscience). Macrophages were resuspended in XF Base DMEM containing 2 mM glutamine, and seeded in a Seahorse Bioscience 96-well plate at the density of 4 10^4^ cells per well. After 3 h, cells were treated with medium or LPS + IFN-γ, and incubated for 24 h at 37°C in an atmosphere of 5% CO_2_.

Glycolytic rate assay was performed. During extracellular flux analysis, cells were sequentially treated with rotenone + antimycin A (final concentration of 10 μM each), followed by 2-deoxy-D-glucose (50 μM, final concentration) and the extracellular acidification rate (ECAR, glycolysis indication) was measured.

Cellular mitochondrial stress test was performed to measure mitochondrial metabolism. Cells were sequentially treated with 2 μM oligomycin A (OA), 1 μM FCCP + 1 mM pyruvate, and 1 μM rotenone + antimycin A to measure the oxygen consumption rate (OCR, mitochondrial respiration indication) and to calculate ATP production (23).

### Statistical analysis

Statistical significance was analyzed by a two-tailed unpaired *t*-test or one-way ANOVA followed by Tukey’s *post hoc* multiple comparison test using Prism software (version 8, GraphPad, San Diego, CA). The survival of mice was tested by Kaplan-Meier analysis. A *p* value <0.05 was considered statistically significant. Each experiment was repeated as indicated in the figure legends; representative results are shown.

## Results

### Drp1 regulates the proinflammatory responses in innate immunity and contributes to the pathology of endotoxin-induced septic shock

**Figure 1:**
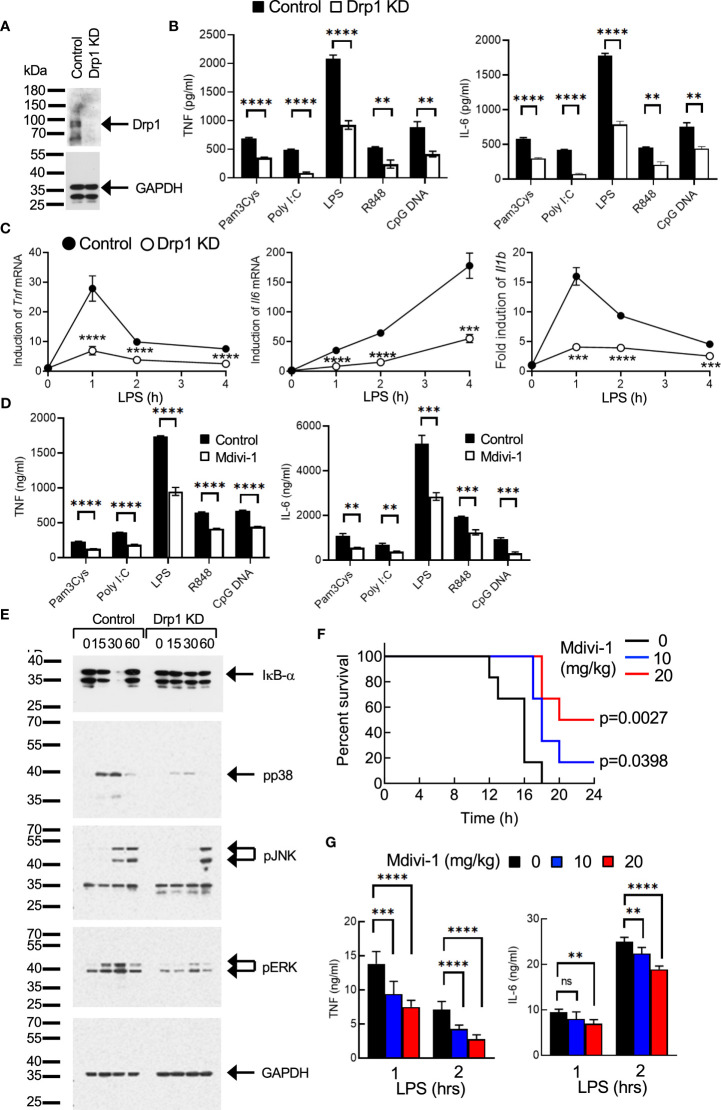
Drp1 regulates the proinflammatory responses in macrophages. **(A–C)**. **(A)** Control or Drp1 KD iBMDMs were **(B)** stimulated with medium or TLR ligands such as Pam3Cys (5 μg/ml), poly I:C (25 μg/ml), LPS (100 ng/ml), R848 (2 μg/ml), or CpG DNA (5 μg/ml). After 24 hours, TNF or IL-6 levels were measured by ELISA (n=4). **(C)** Control or Drp1 KD iBMDMs were treated with LPS (100 ng/ml), and total RNAs were collected at the indicated times to analyze the induction of cytokine mRNAs by qPCR (n=4). **(D)** Peritoneal macrophages were incubated with vehicle (control) or Mdivi-1 (10 μM) for an hour, followed by stimulation with TLR ligands for 24 hours. TNF and IL-6 levels were measured by ELISA (n=4). **(E)** Activation of cell signaling. Control or Drp1 KD iBMDMs were treated with LPS (100 ng/ml), and cell lysates were prepared at the indicated times for Western blot analysis by using the indicated antibodies. GAPDH level was detected as a loading control. **(F, G)** Endotoxin-induced sepsis model. WT mice were injected with vehicle or Mdivi-1 i.p., followed by i.p. injection of LPS (n=9 per group). **(F)** Survival was monitored and **(G)** serum TNF or IL-6 levels were measured by ELISA. Data are shown as mean ± SD. **p<0.01; ***p<0.005; ****p<0.001. n.s., not significant. Representative result of 2-3 repeated experiments is shown.

First, we examined the role of Drp1 in regulating the proinflammatory responses in macrophages. Previously, we generated a macrophage cell line using bone marrow cells from WT mice (21). The immortalized WT bone marrow-derived macrophages (iBMDMs) were infected with lentiviruses encoding control or Drp1 shRNA sequences to knock down Drp1 (**Figure 1A**). Control or Drp1 knockdown (KD) iBMDMs were stimulated with TLR ligands, and the levels of cytokines in culture supernatants were measured after 24 hours. TLR-induced TNF and IL-6 production was reduced significantly in Drp1 KD iBMDMs compared with control cells, indicating that any TLR signaling is involved in the Drp1-mediated inflammatory responses in macrophages (**Figure 1B**). Similarly, the expression of *Tnf*, *Il6*, or *Il1b* mRNAs was induced by LPS treatment in control iBMDMs, whereas KD of Drp1 significantly reduced their expression (**Figure 1C**). We further evaluated the role of Drp1 in macrophages by inhibiting Drp1 activity with Mdivi-1. We found that the production of TNF and IL-6 was also downregulated by inhibition of Mdivi-1 (**Figure 1D**), supporting the role of Drp1 in the regulation of inflammatory responses in macrophages.

Next, we examined how Drp1 regulates the signaling pathways in the TLR-mediated induction of inflammatory responses in macrophages. Degradation of IκB-α, and phosphorylation of p38, JNK, and ERK by LPS treatment were significantly reduced or delayed in Drp1 KD iBMDMs compared with control cells (**Figure 1E**), indicating that Drp1 plays a role in regulating the TLR-proximal signaling pathway in macrophages.

We further examined the role of Drp1 *in vivo* using endotoxin-induced sepsis model in mice. Mice received PBS or Mdivi-1 intraperitoneally, followed by an i.p. injection of LPS. The survival of mice was significantly extended by Mdivi-1 administration, suggesting the proinflammatory role of Drp1 in sepsis (**Figure 1F**). We further observed that serum levels of TNF or IL-6 increased by LPS administration, while Mdivi-1 administration substantially lowered their levels (**Figure 1G**), which indicates the inhibition of Drp1 activity in macrophages reduced the production of proinflammatory cytokines, resulting in the extended survival of mice in sepsis.

Collectively, our results suggest that Drp1 regulates the TLR-mediated signaling pathways for the induction of macrophage inflammatory responses and further contributes to the pathology of endotoxin-induced septic shock in mice, as inhibition of Drp1 extended the survival of mice.

### TLR-dependent PGAM5 activation regulates Drp1-mediated inflammatory responses in macrophages

**Figure 2:**
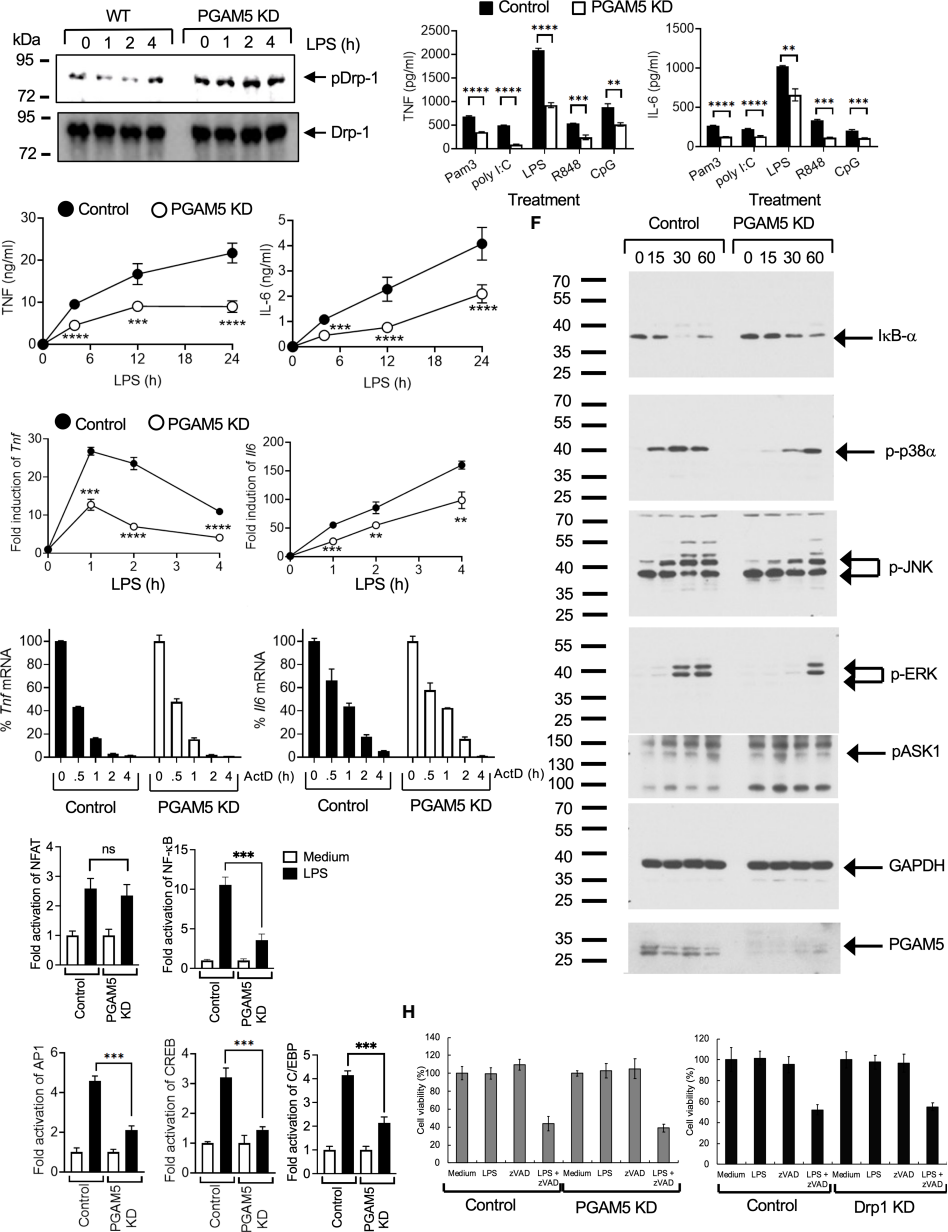
PGAM5 contributes to the induction of proinflammatory responses by regulating the phosphorylation of Drp1 in macrophages. **(A)** Control or PGAM5 KD iBMDMs were stimulated with LPS (100 ng/ml), and the phosphorylation status of Drp1was detected by phospho-Drp1 (S637) Abs. Non-phosphorylated form was detected by Drp1 Abs. **(B–D)**. **(B)** Control or PGAM5 KD iBMDMs were treated with TLR ligands such as Pam3Cys (5 μg/ml), poly I:C (25 μg/ml), LPS (100 ng/ml), R848 (2 μg/ml), or CpG DNA (5 μg/ml) for 24 hours. Culture supernatants were collected to measure TNF or IL-6 levels by ELISA (n=4). **(C)** Control or PGAM5 KD iBMDMs were treated with LPS (100 ng/ml), and culture supernatants were collected at the indicated times to measure TNF or IL-6 concentration by ELISA (n=4). **(D)** Total RNAs were collected at the indicated times to analyze the induction of cytokine mRNAs by qPCR (n=4). **(E)** Control or PGAM5 KD iBMDMs were treated with LPS (100 ng/ml) for 1 (*Tnf*) or 4 (*Il6*) hours and further incubated with ActD (10 μg/ml) for the indicated times. The mRNA level of ActD-unstimulated cells was set as 100%. The relative percentage of Tnf or Il6 levels at each time point was calculated (n=3). **(F)** Control or PGAM5 KD iBMDMs were treated with LPS (100 ng/ml) and the activation of signaling molecules was analyzed by immunoblotting. GAPDH level was detected as a loading control. **(G)** Control or PGAM5 KD iBMDMs were transfected with NF-AT-, NF-κB, AP-1-, C/EBP-, or CREB-driven luciferase plasmids, and treated with LPS for 6 hours after 2 days. Luciferase assay was performed and the activity of pTK-RL was measured as the internal control. Fold induction of luciferase activity was calculated to the untreated cells (n=4). **(H)** Control or PGAM5 KD, and control or Drp1 KD iBMDMs were incubated with medium, LPS (100 ng/ml), zVAD (10 μM), or LPS+zVAD, and cell viability was tested after 24 hours by MTT method (n=6). Data are shown as mean ± SD. **p<0.01; ***p<0.005; ****p<0.001. n.s., not significant. Representative result of 2-3 repeated experiments is shown.

PGAM5 dephosphorylates and activates Drp1, which results in mitochondrial fission and ROS production that cause cellular damage during necrosis (24). Although previous studies showed that Drp1 regulates the expression of cytokines in macrophages and T cells (25, 26), it is still unclear whether PGAM5 directly regulates Drp1 activity in innate immunity. Since our previous study suggests a novel function of PGAM5-Drp1 signaling in the regulation of NKT cell activation during liver inflammation (19), we tested whether Drp1 activation is dependent on PGAM5 activity in innate immunity. To test this, we first examined whether PGAM5 regulates Drp1 phosphorylation. We observed that LPS treatment resulted in the dephosphorylation of Drp1 serine 637 (Ser 637) in control iBMDMs, whereas PGAM5 KD did not show Drp1 dephosphorylation (**Figure 2A**). However, phosphorylation of Drp1 Ser 616 was not detected in macrophages (data not shown). Thus, this result indicates that PGAM5 regulates the phosphorylation status of Drp1 for activation in macrophages.

We further examined the role of PGAM5 in regulating the inflammatory responses of macrophages. Control or PGAM5 KD iBMDMs were stimulated with different TLR ligands. TLR ligand-induced production of TNF or IL-6 was significantly reduced in PGAM5 KD iBMDMs compared with control cells (**Figure 2B**), indicating the role of PGAM5 in macrophage activation. Similarly, TNF or IL-6 production was lower at the early time points after LPS stimulation, and the induction of *Tnf* or *Il6* mRNAs was reduced in PGAM5 KD cells (**Figures 2C, D**), indicating that PGAM5 signaling plays a crucial role in the regulation of inflammatory responses in innate immunity. We further examined whether PGAM5 regulated the stability of inflammatory cytokines in macrophages and found that the rate of *Tnf* or *Il6* mRNA degradation is comparable between control and PGAM5 KD iBMDMs (**Figure 2E**), suggesting that PGAM5 regulates the signaling events that induce the expression of cytokines, while the mRNA stability is not affected by PGAM5.

Next, we compared the TLR-mediated signaling pathways that are crucial for the production of inflammatory cytokines. Degradation of IκB-α, and phosphorylation of p38α, JNK, and ERK were significantly reduced in LPS-treated PGAM5 KD iBMDMs (**Figure 2F**). Additionally, the activation of transcription factors such as NF-κB, AP-1, CREB, and C/EBP by LPS was substantially reduced in PGAM5 KD iBMDMs, while NFAT activation was comparable between control and PGAM5 KD cells (**Figure 2G**). Collectively, our results suggest that PGAM5 regulates TLR-mediated macrophage activation by controlling the activation of downstream signaling pathways.

### PGAM5 signaling in macrophages does not include ASK1 activation and necroptosis

Previous studies have shown that PGAM5 regulates ASK1 activity for the activation of p38α and JNK, and ASK1 induces the activation of the MKK3/6 → p38α pathway in innate immune cells (27–29). Thus, we tested whether PGAM5 regulates the phosphorylation of ASK1. Control or PGAM5 KD iBMDMs were treated with LPS, and ASK1 phosphorylation at Thr 845 was assessed by immunoblotting. ASK1 phosphorylation was increased by LPS treatment, but the level of phosphorylation was comparable between control and KD cells (**Figure 2F**). This result indicated that PGAM5 does not directly regulate ASK1 in the activation of MAP kinase signaling. Therefore, our results ruled out the involvement of ASK1 downstream of PGAM5.

Because PGAM5 is known to regulate the induction of necroptosis, we tested whether necroptosis was involved in PGAM5-mediated macrophage activation. Induction of cell death by inhibition of caspase activity by zVAD in LPS-treated macrophages (necroptosis) was comparable between control and PGAM5 KD iBMDMs (**Figure 2H**), which indicates that PGAM5 does not induce necroptosis during macrophage activation. Additionally, the viability of macrophages was also comparable between control and Drp1 KD cells (**Figure 2H**). Collectively, our results suggest that the regulation of PGAM5-Drp1 activity is an essential aspect of necroptosis-independent TLR-mediated inflammatory responses in innate immune cells.

### PGAM5-Drp1 signaling is dependent on TLR adaptor proteins MyD88 and TRIF to regulate mtROS in macrophages

**Figure 3:**
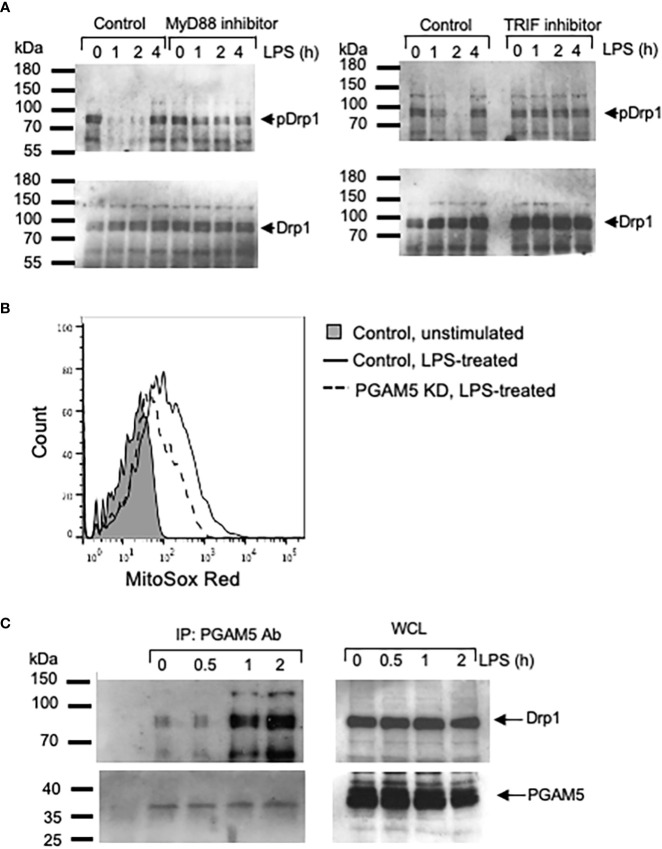
PGAM5 interacts with Drp1 and regulates the production of mtROS in macrophages. **(A)** Peritoneal macrophages from WT mice were incubated with control, MyD88- or TRIF-inhibitory peptide and treated with LPS (100 ng/ml) for indicated times. Dephosphorylation of Drp1 (S656) was analyzed by immunoblotting. **(B)** Control or PGAM5 KD iBMDMs were incubated with MitoSOX and LPS for 12 hours and the intracellular mtROS levels were measured by FACS analysis. **(C)** WT iBMDMs were stimulated with LPS (100 ng/ml). Cell lysates were prepared and immunoprecipitated with anti-PGAM5 Abs. Goat Ig was used as an isotype control. Association of PGAM5 and Drp1, and endogenous PGAM5 and Drp1 levels were detected by blotting with anti-PGAM5 or Drp1 Abs. Representative result of 3 repeated experiments is shown.

Next, we tested whether PGAM5-mediated Drp1 activation is dependent on adaptor proteins such as myeloid differentiation primary response 88 (MyD88) and TIR domain-containing adapter-inducing interferon-β (TRIF) that interact with TLRs upon ligand recognition. To this end, WT peritoneal macrophages were incubated with control, MyD88- or TRIF-inhibitory peptides, followed by LPS or poly I:C stimulation. We observed that LPS- or poly I:C-induced Drp1 dephosphorylation was delayed or reduced by inhibiting MyD88 or TRIF oligomerization (**Figure 3A**), suggesting that TLR-mediated signaling pathways regulate the PGAM5-Drp1-mediated induction of proinflammatory responses in macrophages.

One of the essential functions of mitochondria in macrophages is the generation of ROS (30). In addition to the bactericidal role of mtROS (31, 32), mtROS can also act as a signal that induces the expression of proinflammatory genes by regulating NF-κB and MAPK signaling pathways (8, 33). Thus, we asked whether PGAM5 regulates mtROS generation in macrophages. Stimulation with LPS increased the level of mtROS in control iBMDMs; however, the mtROS level in PGAM5 KD cells was significantly reduced (**Figures 3B**, **S1**), implicating the role of PGAM5 in the generation of mtROS by regulating mitochondrial metabolism in macrophages. Based on this, it is suggested that PGAM5 signaling regulates mtROS production for the expression of inflammatory cytokines.

### LPS treatment increases the association of PGAM5 with Drp1

Activation of TLR signaling regulated the phosphorylation status of Drp1 as PGAM5 dephosphorylated Drp1 by LPS treatment. Thus, we further examined whether PGAM5 interacted with Drp1 in activated macrophages. Association of PGAM5 and Drp1 was detected at a low level in resting macrophages, while LPS treatment increased the formation of PGAM5 and Drp1 (**Figure 3C**), which suggests that MyD88/TRIF-dependent TLR signaling facilitates the formation of PGAM5 and Drp1 complex, leading to the dephosphorylation of Drp1 in macrophages.

### RIPK3 is dispensable in TLR-mediated inflammatory responses

**Figure 4:**
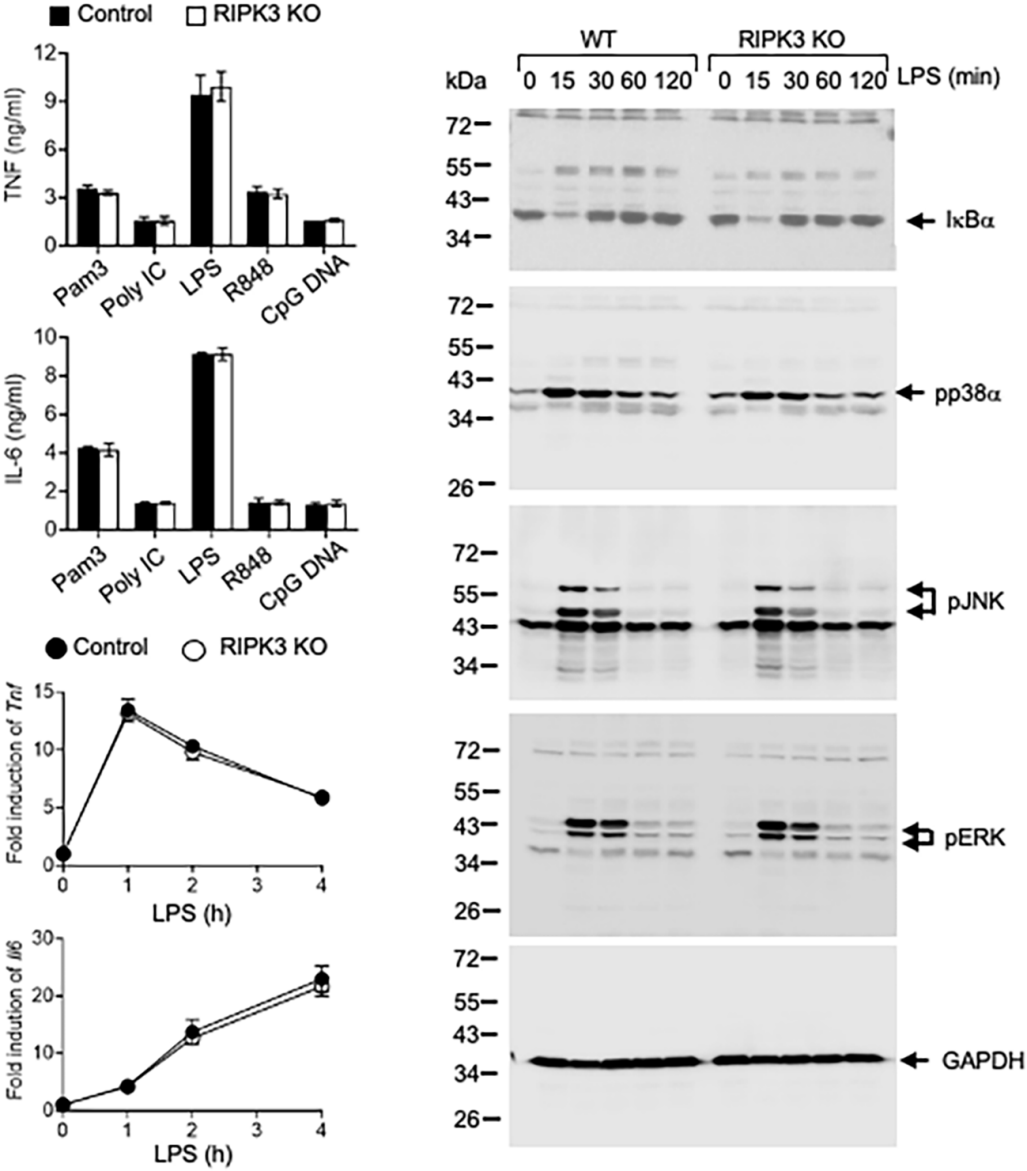
PGAM5-Drp1 signaling is not regulated by RIPK3. **(A)** Peritoneal macrophages from WT or RIPK3 KO mice were treated with TLR ligands such as Pam3Cys (5 μg/ml), poly I:C (25 μg/ml), LPS (100 ng/ml), R848 (2 μg/ml), or CpG DNA (5 μg/ml) for 24 hours. TNF or IL-6 concentrations in culture supernatants were measured by ELISA (n=4). **(B)** WT or RIPK3 KO macrophages were stimulated with LPS (100 ng/ml), and the induction of *Tnf* or *Il6* mRNAs was measured by qPCR analysis (n=4). **(C)** WT or RIPK3 KO macrophages were treated with LPS (100 ng/ml), and the degradation of IκBα and phosphorylation of MAPKs were analyzed by immunoblotting. GAPDH level was detected as a loading control. Representative result of 2-3 repeated experiments is shown.

PGAM5 has been identified as a key regulator of RIPK3-mediated signaling since PGAM5 interacts with the RIPK1/RIPK3 complex after stimulation. We tested whether RIPK3 is involved in TLR-mediated inflammatory responses in innate immunity. Peritoneal macrophages from WT or RIPK3 KO mice were treated with different TLR ligands, and TNF and IL-6 levels were measured. Proinflammatory cytokine levels induced by TLR ligand were comparable between WT and RIPK3 KO macrophages, indicating that RIPK3 does not regulate PGAM5-mediated inflammatory responses in macrophages (**Figure 4A**). Similarly, RIPK3 deficiency did not affect the levels of LPS-induced *Tnf* or *Il6* mRNAs in macrophages (**Figure 4B**).

TLR-mediated signaling pathways were examined. LPS-induced IκB-α degradation and p38α, JNK, and ERK phosphorylation were comparable between WT and RIPK3 KO peritoneal macrophages (**Figure 4C**). Collectively, our data indicates that RIPK3 does not play a role in regulating the induction of inflammatory responses, and PGAM5 is not regulated by RIPK3 activity in macrophages.

### PGAM5-Drp1 signaling contributes to the induction of proinflammatory macrophage phenotype

**Figure 5:**
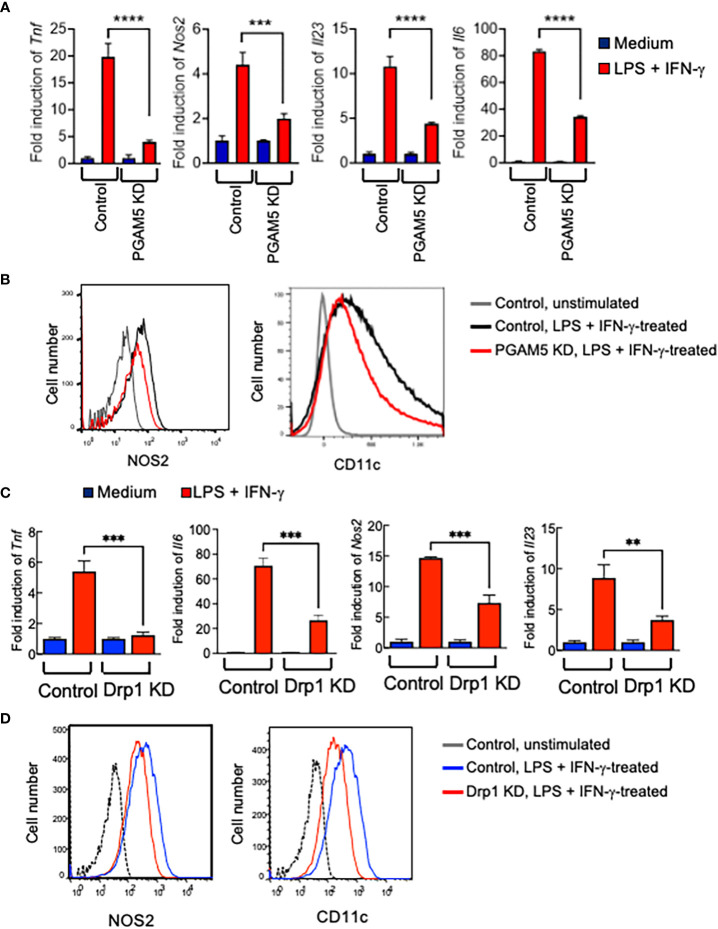
PGAM5-Drp1 promotes the polarization of macrophages toward proinflammatory phenotype. **(A, B)** Control or PGAM5 KD iBMDMs or **(C, D)** control or Drp1 KD iBMDMs were treated with medium or LPS (100 ng/ml) + IFN-γ (100 ng/ml) for 24 hours. **(A, C)** Total RNAs were prepared and the expression of pro-inflammation genes was analyzed by qPCR (n=4) and **(B, D)** the expression of CD11c and NOS2 was analyzed by FACS. Data are shown as mean ± SD. **p<0.01; ***p<0.005; ****p<0.001. Representative result of 2-3 repeated experiments is shown.

High plasticity and adaptation to various stimulatory conditions are the hallmark of macrophages. Emerging evidence shows that contrasted metabolic activities determine the proinflammatory or anti-inflammatory phenotypes of macrophages (4–6). We further examined whether PGAM5-Drp1 signaling plays a role in the determination of macrophage phenotype. Classically activated and pro-inflammatory M1 macrophages are induced by LPS and IFN-γ, while IL-4 treatment increases the induction of alternatively activated M2 macrophages that express the anti-inflammatory genes *in vitro* (34). To test the role of PGAM5 in the induction of M1 macrophages, control or PGAM5 KD iBMDMs were incubated with LPS + IFN-γ, and the expression of proinflammatory markers was examined. LPS-induced expression of *Tnf*, *Nos2*, *Il23*, or *Il6* in control cells was reduced significantly by KD of PGAM5 (**Figure 5A**), indicating that PGAM5 regulates the promotion of the proinflammatory phenotype of macrophages. Moreover, flow cytometry analysis indicated that the expression levels of NOS2 and CD11c, which are markers of proinflammatory macrophages, were lower in PGAM5 KD iBMDMs compared with control cells (**Figures 5B**, **S1**). Furthermore, Drp1 also regulated the induction of proinflammatory responses in macrophages, as the expression of proinflammatory genes was significantly reduced in LPS + IFN-γ-treated Drp1 KD iBMDMs compared with control cells (**Figures 5C, D**, **S1**). Collectively, it is suggested that PGAM5-Drp1 signaling contributes to the promotion of the proinflammatory macrophage phenotype.

### PGAM5 promotes the metabolic reprogramming of proinflammatory macrophages

High plasticity and adaptation to various stimulatory conditions are the hallmarks of macrophages. Emerging evidence shows that contrasted metabolic activities determine the proinflammatory M1 macrophages or anti-inflammatory M2 phenotypes of macrophages (4–6). Therefore, we further examined whether PGAM5 regulates the metabolic reprogramming of macrophages that results in the induction of a proinflammatory phenotype.

**Figure 6:**
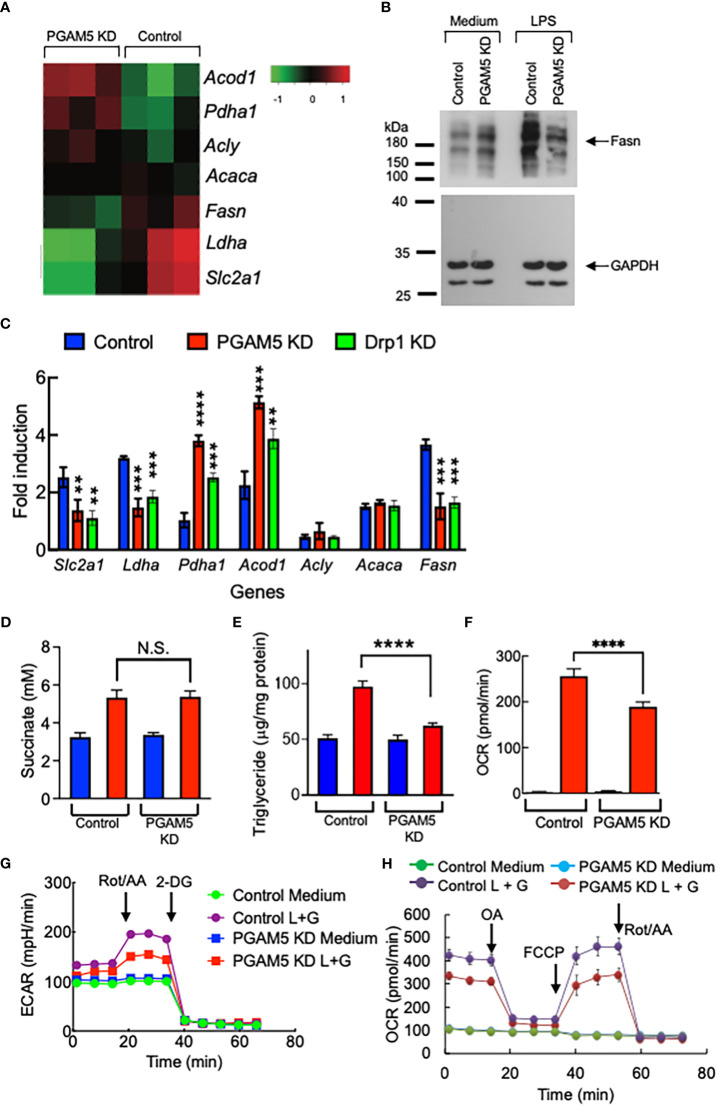
PGAM5 regulates the metabolic reprogramming of macrophages. **(A)** RNA-seq analysis of metabolism genes. Control or PGAM5 KD iBMDMs were treated with LPS for 4 hours and total RNA samples were prepared for RNA-seq analysis. **(B)** Control or PGAM5 KD iBMDMs were treated with LPS (100 ng/ml) for 24 hours and the expression of Fasn was detected by immunoblotting. The level of GAPDH was detected as a loading control. **(C)** Control, PGAM5 KD, or Drp1 KD iBMDMs were treated with LPS for 4 hours, and metabolism gene expression was analyzed by qPCR (n=4). **(D, E)** Control or PGAM5 KD iBMDMs were treated with LPS (100 ng/ml) for 24 hours and intracellular levels of succinate and TG were measured (n=4). **(F–H)** Real-time metabolic analysis (n=4). Control or PGAM5 KD iBMDMs were treated with LPS + IFN-γ for 24 hours, and glycolytic rate or mitochondrial stress assays were performed to measure the changes in **(F)** ECAR, **(G)** OCR, or **(H)** ATP production. Data are shown as mean ± SD. **p<0.01; ***p<0.005; ****p<0.001. N.S., not significant. Representative result of 2-3 repeated experiments is shown.

First, we examined whether the expression of metabolism genes is regulated by PGAM5 in macrophages by RNA-seq analysis. By comparing the data from LPS-treated control or PGAM5 KD iBMDMs, we found that LPS-induced expression of glucose transporter 1 (Glut1, *Slc2a1*), lactate dehydrogenase A (*Ldha*) and fatty acid synthase (*Fasn*) was significantly reduced by KD of PGAM5, while that of ATP citrate synthase (*Acly*), and acetyl-CoA carboxylase (*Acaca*) was comparable between control and PGAM5 KD cells. However, pyruvate dehydrogenase E1 subunit α 1 (*Pdha1*) and aconitate decarboxylase 1 [*Acod1*, or immunoresponsive gene 1 (*Irg1*)] expression by LPS was substantially increased in PGAM5 KD macrophages (**Figures 6A, B**). We further confirmed the expression of metabolism genes between LPS-treated control, PGAM5 KD, and Drp1 KD iBMDMs was compared by qPCR analysis (**Figure 6C**). This result indicates that glycolysis and fatty acid synthesis are tightly regulated by the mitochondrial phosphatase PGAM5 for the metabolic reprogramming of macrophages to a proinflammatory phenotype.

Upon LPS stimulation of macrophages, mitochondrial TCA cycle remodeling is accompanied, which results in TCA metabolite accumulation, including succinate, which plays a critical role in inflammatory cytokine production (35–37). We found that LPS-induced succinate production was comparable between control and PGAM5 KD macrophages (**Figure 6D**). Although glycolysis was reduced, *Acod1* expression was increased in PGAM5 KD macrophages. Another metabolic feature of LPS-stimulated proinflammatory macrophages is fatty acid synthesis, leading to the accumulation of lipids in the form of triacylglycerol (TG) (2, 38). We found that LPS stimulation increased the concentration of TG in control macrophages, but not in PGAM5 KD cells (**Figure 6E**), which is consistent with the finding that the expression of *Fasn* is reduced in PGAM5 KD macrophages (**Figure 6B**).

To further test the role of PGAM5 in regulating cell metabolism, glycolysis and mitochondrial metabolism were analyzed using a Seahorse XF analyzer. By comparing the extracellular acidification rates (ECAR) between LPS +IFN-γ-treated control and PGAM5 KD iBMDMs, we found that KD of PGAM5 reduced the rate of glycolysis in macrophages (**Figure 6F**). Additionally, we found that PGAM5 also alters mitochondrial oxidative phosphorylation (OXPHOS) as the oxygen consumption rates (OCR) were significantly lower in PGAM5 KD iBMDMs than control cells (**Figure 6G**). Furthermore, ATP production induced by LPS + IFN-γ was significantly reduced in PGAM5 KD macrophages compared with control cells (**Figure 6H**).

**Figure 7:**
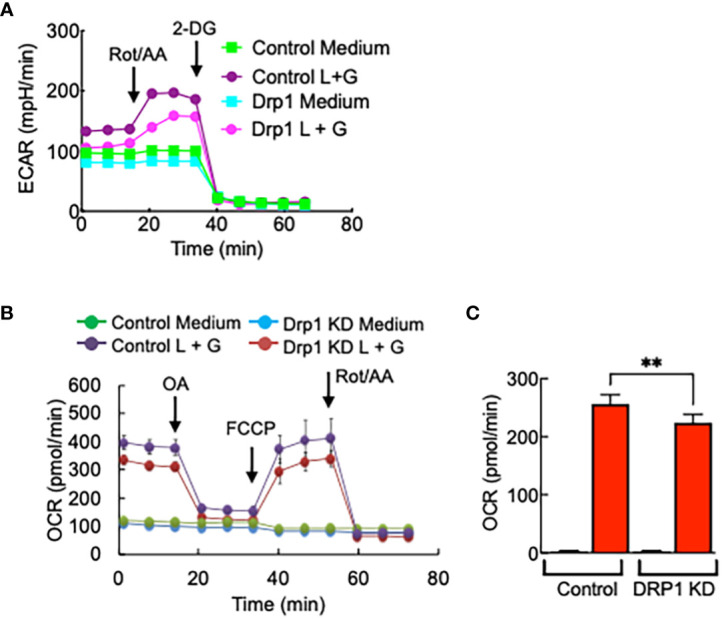
Drp1 regulates macrophage metabolism. Control or Drp1 KD iBMDMs were treated with LPS (100 ng/ml) + IFN-γ (100 ng/ml) for 24 hours, and glycolytic rate assay or mitochondrial stress assay were performed to measure the changes in **(A)** ECAR, **(B)** OCR, or **(C)** ATP production (n=3). Data are shown as mean ± SD. **p<0.001. Representative result of 2-3 repeated experiments is shown.

Glycolysis rate, mitochondrial respiration, and ATP production were reduced in Drp1 KD iBMDMs compared with control cells, although the degree of reduction is not as significant as that of PGAM5 KD cells (**Figures 7A–C**).

Collectively, these data indicate that PGAM5 promotes the metabolic reprogramming of proinflammatory macrophages by regulating glycolysis and mitochondrial metabolism, which further contribute to fatty acid synthesis in macrophages and the induction of proinflammatory responses in macrophages.

## Discussion

Mitochondria play a critical role in the activation, differentiation, and survival of immune cells. Microbial or viral infection or inflammatory stimuli may lead to changes in mitochondrial metabolism and physiology, resulting in the metabolic switching of macrophage activation (8, 39). In this study, we demonstrated how the mitochondrial phosphatase PGAM5 regulates proinflammatory responses in macrophages.

Dynamic and continuous fusion and fission of mitochondria are critical for maintaining their shape, size, and intracellular distribution, which is regulated by dynamin-related GTPases such as Drp1 (10, 40). Previous studies demonstrated that LPS-induced activation of Drp1 regulates the mitochondrial fission and subsequent inflammatory responses of macrophages and contributes to the promotion of vascular remodeling after injury (14, 15). Furthermore, PKCδ-dependent phosphorylation of Drp1 or Stat2-dependent phosphorylation of Drp1-dependent mitochondrial mass increase might be an upstream mechanism that regulates the induction of proinflammatory responses in macrophages (16, 17), suggesting that Drp1 plays a crucial role in connecting the changes in mitochondrial dynamics and innate immunity. In contrast, a study showed that KD of Drp1 augmented the activation of NLRP3 inflammasome activation without changing mtROS production or mitochondrial damage (41).

We also examined the mechanism of Drp1-mediated regulation of macrophage activation. Consistent with other studies, we observed that TLR-dependent inflammatory responses of macrophages were significantly reduced by KD or inhibition of Drp1, supporting the finding about the role of Drp1 in innate immune cells. Furthermore, macrophages Drp1 regulates the activation of NF-κB and MAPK pathways in TLR signaling and contributes to the promotion of vascular remodeling after injury (15). Similarly, we found that pharmacological inhibition of Drp1 by Mdivi-1 alleviated the pathology of endotoxin-induced septic shock in mice, suggesting the proinflammatory role of Drp1 in the development of inflammatory diseases and implicating its potential as a target of anti-inflammation treatment.

In our previous study, we found that PGAM5 regulated the phosphorylation of Drp1 in NKT cells, contributing to liver inflammation and anti-tumor immunity (19). Thus, we tested whether PGAM5 is an upstream molecule that directly regulates Drp1 activity in macrophages. Previous studies demonstrated the role of PGAM5 in innate immunity. PGAM5 regulates the activity of inflammasome and caspase 1 in BMDMs and contributes to IFN-β production in response to viral infection by promoting the TBK1 (TANK-binding kinase 1)/IFN regulatory factor 3 (IRF3) signaling pathway (42, 43). Knockdown of PGAM5 in macrophages significantly reduced the LPS-induced expression of inflammatory cytokines, indicating that PGAM5 regulates the activation of TLR signaling in macrophages. Moreover, the induction of the proinflammatory response and the activation of NF-κB and MAPKs signaling were reduced or delayed in PGAM5 knockdown macrophages treated with all TLR ligands we tested (TLR2, 3, 4, 7, 9 ligands). However, activation of ASK1 was not regulated by PGAM5 in macrophages, although PGAM5 regulated the activation of ASK1 in NKT cells, indicating that the downstream signaling pathway regulated by PGAM5 in macrophages differs from that in NKT cells. Collectively, our results indicate that PGAM5 is involved in the common pathway of TLR signaling in macrophages. Therefore, PGAM5 may play a crucial role in innate immunity as a regulator with a broad spectrum of TLR responses. Additionally, our preliminary results suggest the role of mitochondria in providing a platform for host protection against microbial infection and regulation of inflammation.

Activation of Drp1 is determined by the phosphorylation status of Ser residues, as phosphorylation at Ser 616 and dephosphorylation at Ser 637 implicate the Drp1 activation. Previous studies showed that Stat2 phosphorylates Drp1 at serine 616 for Drp1-mediated mitochondrial fission (44) and that protein kinase C (PKC) δ regulates the mitochondrial fission by phosphorylating Drp1 at Ser 616 (16). Additionally, phosphorylation of Ser 637 in HEK 293T cells is mediated by PKA (44). Furthermore, a study indicated that LPS induced Drp1 phosphorylation at Ser 616 immediately, followed by sustained Drp1 dephosphorylation at Ser 673 in bone marrow-derived macrophages (14), while only dephosphorylation of Drp1 at Ser 637 was detected in LPS-treated macrophages in our study. The exact mechanism of Drp1 modification and the effect of Drp1 phosphorylation on its activation or inhibition are likely dependent on cell types and cellular signals.

A previous study showed that the phosphorylation status of Ser 637 in Drp1 does not play a role in the recruitment of Drp1 to mitochondria. However, we found that LPS treatment regulates the phosphorylation status of Drp1 Ser 637 and the association of Drp1 and PGAM5 in macrophages, implicating that the Ser 637 residue in Drp1 might be essential for the recruitment of Drp1 to mitochondria. In our experiment, macrophages were stimulated, and the association of endogenous Drp1 and PGAM5 was detected by immunoprecipitation, while the study used the overexpression system of WT and mutant Drp1 plasmids in HEK 293 cells, resulting in a discrepancy in the requirement of Ser 637 in Drp1. Additionally, regardless of the phosphorylation status, it is suggested that Drp1 is recruited to mitochondrial PGAM5, followed by Ser 637 phosphorylation of Drp1 in LPS-treated macrophages.

Initiation of TLR signaling triggers the recruitment of receptor-proximal adaptors such as MyD88 and TRIF for the activation of downstream signaling that induces inflammatory responses in innate immune cells. Dephosphorylation of Drp1 was reduced or delayed by treating macrophages with MyD88- or TRIF-specific inhibitory peptides, indicating that the PGAM5-Drp1-mediated inflammatory response is dependent on the TLR-proximal signaling pathway. A previous study also suggested an involvement of MyD88 in LPS-mediated dephosphorylation of Drp1 (S656) (14). Mitochondrial ROS can act as signaling molecules to regulate the expression of cytokine genes (25, 26), while the dephosphorylation of Drp1 by activated PGAM5 induces mtROS generation (13, 24). In addition to the previous finding that Drp1 regulates the production of mtROS in macrophages (15), PGAM5 KD reduced mtROS levels in LPS-treated macrophages, suggesting the role of PGAM5-Drp1-mediated signaling in mtROS production for the induction of inflammatory responses. In our previous study, however, PGAM5 did not regulate the production of mtROS in NKT cells, which supports the notion that PGAM5-Drp1 signaling regulates the downstream pathways in a cell-type manner. Furthermore, it seems that the association of PGAM5 and Drp1 plays a crucial role in the inflammatory responses of macrophages, as PGAM5 forms a complex with Drp1 on LPS stimulation.

Previous studies showed that RIPK3 is dispensable for signaling pathways mediated by T cell and B cell receptors, TNFR, and TLR, and that RIPK3 does not regulate PGAM5 activity in macrophage inflammasome activation (42, 45). Our results also showed that the TLR-proximal signaling pathways for the expression of inflammation genes are not regulated by RIPK3.

Mitochondria play a crucial role in regulating cell metabolism, which is closely associated with the determination of the inflammatory phenotype of macrophages. Accordingly, consistent with the expression of proinflammatory cytokines, PGAM5 promoted M1 polarization of macrophages, as the expression of M1 markers was significantly reduced in PGAM5 KD macrophages, implicating that PGAM5 regulates the metabolic reprogramming of macrophages. In macrophages, LPS stimulation induced the expression of genes involved in glycolysis and fatty acid synthesis, on which M1 macrophage polarization relies. Expression levels of *Slc2a1* and *Ldha*, which are essential for glycolysis in macrophages, were lower in PGAM5 KD macrophages than in control cells. Accordingly, the rate of glycolysis determined by ECAR was significantly reduced in PGAM5- or Drp1-KD macrophages, supporting the finding that PGAM5-Drp1 signaling regulates glycolysis in macrophages. However, despite the decrease in glycolysis, the intracellular succinate level was not affected by the KD of PGAM5. We found that the expression of *Acod1* was higher in PGAM5 KD macrophages than control cells, leading to the inhibition of succinate dehydrogenase (SDH) for the accumulation of succinate in macrophages (46). ACOD1 promotes the production of itaconate by cis-aconitate decarboxylation in the mitochondrial matrix (47), leading to the metabolic switching of pr-inflammatory macrophages to an anti-inflammatory phenotype to facilitate tissue repair by limiting the proinflammatory conditions in macrophages (48, 49). Since *Acod1* expression was increased in PGAM5 KD macrophages, it is implicated that PGAM5-Drp1 signaling negatively regulates the expression of *Acod1* for the induction of proinflammatory responses in macrophages. However, the detailed mechanism of PGAM5-dependent ACOD1 regulation is not clear. Furthermore, PGAM5-Drp1 signaling regulates mitochondrial metabolism in macrophages, as PGAM5 KD macrophages exhibit dysfunctional mitochondrial respiration and a reduction of ATP production in addition to reduced mtROS production. Thus, it is suggested that PGAM5-Drp1 signaling regulates mtROS production reduction, oxygen consumption inhibition, and ATP reduction (50), leading to the increase in fatty acid synthesis (51).

Collectively, our results suggest that TLR-MyD88/TRIF-dependent PGAM5-Drp1 signaling regulates the induction of proinflammatory responses in macrophages by regulating the metabolic reprogramming of macrophages.

## Data availability statement

RNA-Seq data have been deposited at GEO and are openly available in NCBI GEO at https://www.ncbi.nlm.nih.gov/geo, reference number GSE235306.

## Ethics statement

The animal study was approved by Institutional Animal Care and Use Committee, The Scripps Research Institute. The study was conducted in accordance with the local legislation and institutional requirements.

## Author contributions

B-RB and HM performed experiments. YK designed and performed the experiments and wrote the manuscript. All authors contributed to the article and approved the submitted version.
