# Supplementary material for: Mitochondrial PGAM5−Drp1 signaling regulates the metabolic reprogramming of macrophages and regulates the induction of inflammatory responses
Source: Front Immunol. 2023 Sep 12;14:1243548. doi: 10.3389/fimmu.2023.1243548 (PMC10523165; doi:10.3389/fimmu.2023.1243548)
Supplement: Supplementary file 1 [file Presentation_1.pdf]

Supplementary Figure S1

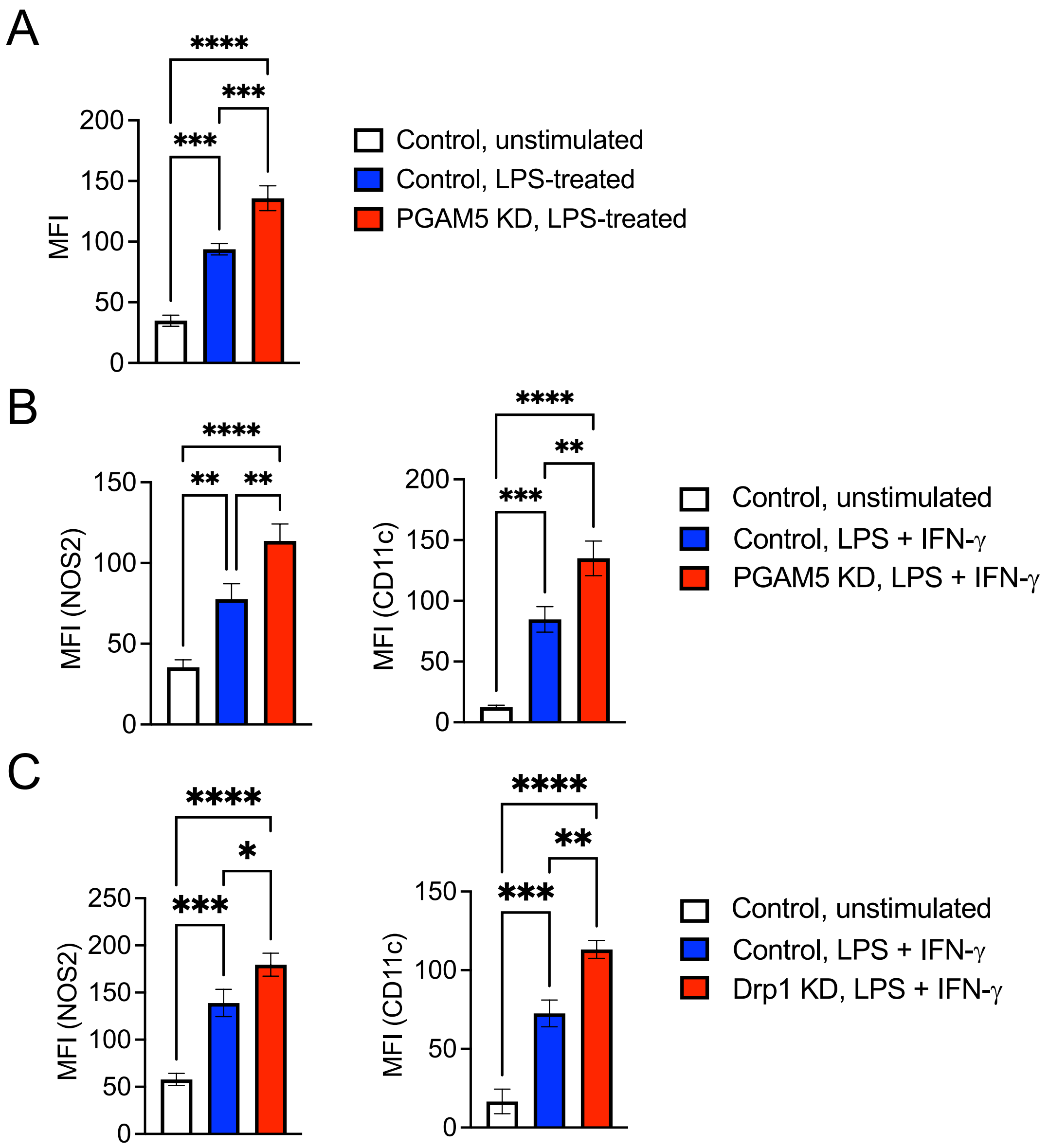

Figure S1. Flow cytometry of mtROS production and M1 macrophage polarization. Mean fluorescence intensity (MFI) of (A) mtROS levels in LPS-treated control or PGAM5 KD macrophages, and (B&C) the expression of NOS2 and CD11c in LPS/IFN-γ-treated (B) control or PGAM5 KD or (C) control or Drp1 KD macrophages are shown. Data are shown as mean ± SD. N=3. *, $p<0.05$; **, $p<0.01$; ***$p<0.005$; ****$p<0.001$.
